# Monitoring the spread of meticillin-resistant *Staphylococcus aureus* in The Netherlands from a reference laboratory perspective

**DOI:** 10.1016/j.jhin.2016.02.022

**Published:** 2016-08

**Authors:** T. Donker, T. Bosch, R.J.F. Ypma, A.P.J. Haenen, W.M. van Ballegooijen, M.E.O.C. Heck, L.M. Schouls, J. Wallinga, H. Grundmann

**Affiliations:** a University Medical Center Groningen, University of Groningen, Groningen, The Netherlands; b National Institute for Public Health and the Environment (RIVM), Bilthoven, The Netherlands

**Keywords:** Meticillin-resistant *Staphylococcus aureus*, Epidemiology, Algorithm, Surveillance

## Abstract

**Background:**

In The Netherlands, efforts to control meticillin-resistant *Staphylococcus aureus* (MRSA) in hospitals have been largely successful due to stringent screening of patients on admission and isolation of those that fall into defined risk categories. However, Dutch hospitals are not free of MRSA, and a considerable number of cases are found that do not belong to any of the risk categories. Some of these may be due to undetected nosocomial transmission, whereas others may be introduced from unknown reservoirs.

**Aim:**

Identifying multi-institutional clusters of MRSA isolates to estimate the contribution of potential unobserved reservoirs in The Netherlands.

**Methods:**

We applied a clustering algorithm that combines time, place, and genetics to routine data available for all MRSA isolates submitted to the Dutch Staphylococcal Reference Laboratory between 2008 and 2011 in order to map the geo-temporal distribution of MRSA clonal lineages in The Netherlands.

**Findings:**

Of the 2966 isolates lacking obvious risk factors, 579 were part of geo-temporal clusters, whereas 2387 were classified as MRSA of unknown origin (MUOs). We also observed marked differences in the proportion of isolates that belonged to geo-temporal clusters between specific multi-locus variable number of tandem repeat analysis (MLVA) clonal complexes, indicating lineage-specific transmissibility. The majority of clustered isolates (74%) were present in multi-institutional clusters.

**Conclusion:**

The frequency of MRSA of unknown origin among patients lacking obvious risk factors is an indication of a largely undefined extra-institutional but genetically highly diverse reservoir. Efforts to understand the emergence and spread of high-risk clones require the pooling of routine epidemiological information and typing data into central databases.

## Introduction

Occurrence of meticillin-resistant *Staphylococcus aureus* (MRSA) in hospitals differs markedly between countries.^1^ The low MRSA rates in The Netherlands have conventionally been attributed to the so-called Dutch search-and-destroy policy, which stipulates that all patients who have had MRSA in the past or who are regarded at high risk of being colonized or infected are screened on admission and treated in strict isolation until screening results become available.^2^, ^3^ Despite these efforts, hospitals in The Netherlands are not free of MRSA. Time and again, MRSA is isolated from hospitalized patients without obvious risk factors after being admitted for more than two days. This leads to extensive screening of contact patients and possibly even hospital staff, which can be rather disruptive.

Patients with these unexpected MRSA findings pose an epidemiological and public health challenge. They either represent cases of primary introduction, which are regarded as MRSA of unknown origin (MUO), or are the result of unobserved secondary spread.^4^ The first would be suggestive of a tip of the iceberg whereby the frequency with which MUOs are isolated in hospitals would be a reflection of an undetermined extra-institutional reservoir, the second an indication of hidden intra- or inter-institutional transmission chains.

Conventional hospital-based investigational epidemiology has its limitations as it can only link cases with an apparent epidemiological association, for instance if patients had shared a room. Conversely, data available at national public health institutes or reference laboratories contain too little information to identify these obvious epidemiological links. However, molecular typing data, and information about the location and time of isolation, may provide sufficient detail to address some of the above-mentioned challenges.

By combining data available at the Dutch staphylococcal reference laboratory at the National Institute for Public Health and the Environment (RIVM) we map the distribution of multi-locus variable number of tandem repeat analysis (MLVA) types over time and space and estimate the size and clonal composition of institutional and multi-institutional MRSA clusters. We also determine the number and genetic diversity of MUOs as an estimate of the frequency of primary hospital introductions by patients without the obvious risk factors of MRSA carriage. Using the results of these analyses, we draw conclusions about the existence of unknown reservoirs outside of hospitals and the differential transmissibility of MRSA clones within healthcare settings.

## Methods

### Data and algorithm

#### MRSA surveillance

Primary MRSA isolates from patients admitted to Dutch hospitals are routinely sent to the RIVM by all microbiological laboratories in The Netherlands for reference typing. Each isolate is investigated by MLVA, staphylococcal protein A (spa) typing and mecA and lukS lukF polymerase chain reaction (PCR) as part of the standard reference service.^5^ The hospitals are asked to complete a questionnaire about epidemiological metadata for each isolate. This questionnaire includes questions about the anatomical site of isolation, demographics of the patient, the reason for sampling, and whether the patient belonged to one of the risk groups defined by the Workgroup Infection Prevention.^3^ We used data collected between 2008 and 2011 (four years), and included only the isolates for which information for the following variables was available: date of isolation, MLVA type, postal code of patients' residence, and submitting laboratory. Duplicate isolates from the same patient, same laboratory, and same year were excluded. Typing data partitioned at the level of MLVA clonal complex (MC) were used; these essentially overlap with MLST clonal complexes with the same number.^5^ Because of the large number of isolates belonging to MC398, and because the algorithm is computationally intensive, we only used data from 2009 for the MC398 clonal complex.

#### Cluster algorithm

To identify clusters of isolates in the MRSA surveillance database, a non-parametric clustering algorithm proposed by Ypma *et al.* was used. In short, this algorithm counts the number of isolates between any two samples for each of the available variables, to calculate the combined distance between them.^6^ The isolates are then joined to form a ‘transmission tree’ of related isolates, based on the calculated distances. Starting with the entire transmission tree as one possible cluster, the weakest link in this cluster, i.e. the link with the longest distance between two isolates, is recorded together with the size of the cluster. The cluster is subsequently split up by this weakest link, resulting in two new possible clusters, two single isolates, or one single isolate and one possible cluster. This splitting is repeated on each possible cluster until all links are removed and only single isolates are left, at which point (*N* ‒ 1) possible clusters are recorded together with their weakest link, where *N* is the number of isolates. After all combinations of cluster size and the strength of the weakest link in the cluster are recorded, they are tested against 1000 randomly generated transmission trees to assess whether its weakest link was stronger than would be expected for a cluster of the same size under random assumption.

### Permutation test

A permutation test was performed to assess whether the weakest link in each observed cluster was stronger than could be expected if all isolates were epidemiologically unrelated (in which case they should be randomly distributed over the variables). For each of the variables, a random order of isolates was chosen, thus creating random combinations of time, location, and genetics per isolate. For each permuted dataset, the cluster algorithm was performed as described above, keeping track of all weakest links for each possible cluster size. The strongest of these weakest links is as close as isolates are expected to be at random, depending on the size of cluster they are in, and is recorded. This process was repeated on 1000 permutation datasets. The resulting distribution of link strengths for each cluster size was then used to test the observed cluster size‒weakest link combinations.

### Distance measures

For each of the variables, a distance metric needs to be defined. For the date of isolation this is simply the difference in days between the dates, and for residence postal code it is the distance between the centroids of the postal code areas. For the MLVA data, the number of VNTR loci difference between the isolates was used (range: 0–8). For the distance measure between laboratories, we used the referral distance between the main hospitals they serve. This is the shortest path between two hospitals through the national patient referral network, formed by the exchange of patients between hospitals. The shortest paths between all hospitals were determined using a simulated patient referral network (see Supplementary data).^7^

### Epidemiological validation

The cluster algorithm was applied using the following variables: isolation date, postal code of patients' residence, and MLVA type. In a subsequent analysis, postal code of patients' residence was replaced by position of healthcare institution within the Dutch referral network. For each MLVA clonal complex, we recorded the number of isolates that form geo-temporal clusters, the total number of clusters, and the number of clusters that included isolates from more than one hospital.

In order to test the robustness of the algorithm, we assessed the influence of spurious connections on the clustering by repeating the analysis on randomly chosen subsets of the data. We selected 90% of the isolates and repeated the cluster algorithm and permutation test. This jack-knife process was repeated 1000 times. The distribution of the percentage clustered isolates was compared with the observed point-estimate, using the entire dataset. We also performed the algorithm on the isolates subdivided per year for the MLVA complexes that contained >100 isolates per year (i.e. MC5, MC8, MC22, and MC45) to test the influence of using data collected over shorter time-frames.

Certain risk categories defined by the Dutch Working Group for Infection Prevention (WIP) such as patients who had been in contact with a known MRSA carrier are more likely associated with nosocomial transmission, and therefore isolates from these patients are more likely to form clusters.^3^ Conversely, if patients were screened because they had previously been hospitalized outside The Netherlands, the isolates may be regarded as introductions, and not likely to form a cluster. Isolates that had multiple conflicting WIP categories attached or that were recorded as isolates without accompanying WIP categories were marked as conflicting cases. We further measured the proportion of clustered isolates for isolates without apparent risk factors: since no epidemiological information was submitted or no known risk factors were identified, no expectation could be formulated. We compared the odds of an isolate belonging to a cluster, stratified by WIP risk category, to test whether the results coincided with the expectations based on their epidemiological profile. Next to the provided epidemiological information, we assessed whether isolates that carried the Panton‒Valentine Leukocidin (PVL) gene, which is associated with community acquisition of MRSA, were more often found in isolates that did not cluster.

## Results

### MRSA surveillance

**Figure 1:**
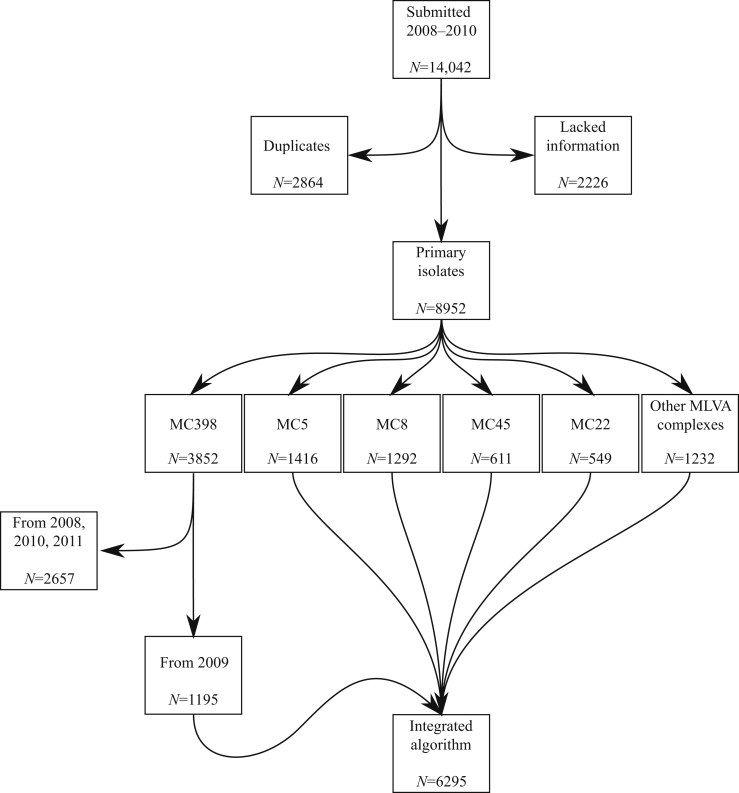
Isolates included in the study. Of the 14,042 isolates submitted to the Dutch National Institute for Public Health and the Environment, 5090 isolates were excluded (2864 duplicates and 2226 lacking information). A further 2657 MC398 isolates were excluded to reduce computational time, yielding a total of 6295 isolates used in the integrated algorithm.

Between 2008 and 2011, a total of 14,042 MRSA isolates were submitted to the RIVM for reference confirmation and typing. Of these, 2864 were duplicate isolates and 2226 lacked information about the patient's place of residence and were excluded from the analysis. Molecular typing grouped 7720 (86%) isolates into five dominant MLVA clonal complexes (MC, Figure 1; Supplementary Table I). The remaining 1232 isolates belonged to an additional 11 MCs. MC398 contained most isolates (3852, 43%), coinciding with sequence type (ST)398 which represents livestock-associated MRSA in The Netherlands.^8^ Since the integrated cluster algorithm scales exponentially with the number of isolates, inclusion of all MC398 isolates became computationally too intensive and only MC398 isolates for 2009 were included in the current study, yielding a total of 6295 isolates.

### Clusters

**Figure 2:**
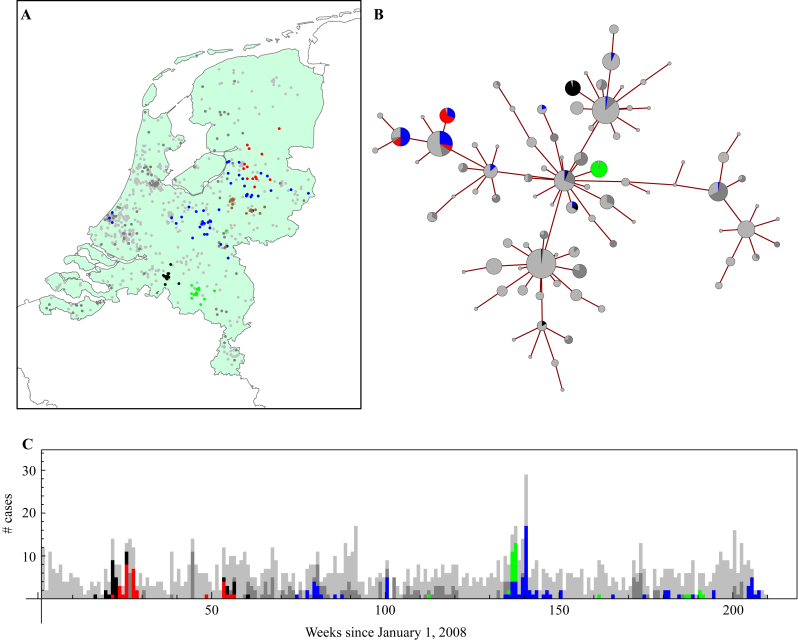
Data sources for cluster detection in meticillin-resistant *Staphylococcus aureus* isolates in The Netherlands 2008 to 2010, MLVA (multi-locus variable number of tandem repeat analysis) complex 8. (A) The postal code of the patients' home addresses, (B) the MLVA complex, here shown as a minimum spanning tree, with each circle showing a single MLVA type (size increasing with number of isolates), and (C) time at which the sample was isolated, here shown in weekly aggregate numbers. Light grey denotes isolates outside clusters; blue, red, green, and black show the isolates present in the four largest clusters. The other clustered isolates are shown in dark grey. Whereas each of the sources shows weak clustering pattern, the three sources, taken together, reveal considerably stronger clustering pattern.

**Figure 3:**
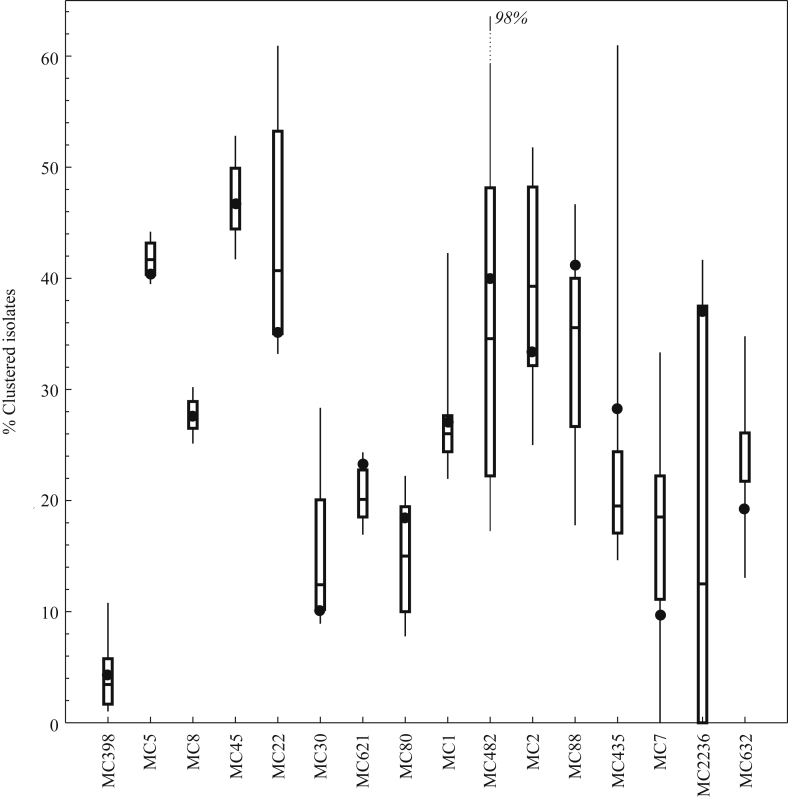
The proportion of isolates that is part of a cluster according to the algorithm. Dots show the point estimate; box plots (median, interquartile range, 95% interval) include jack-knife estimates for 90% of the isolates. Frequently occurring hospital-associated meticillin-resistant *Staphylococcus aureus* (MRSA) strains (MC5, MC45, MC22) show higher proportions of clustering than community (MC8)- or livestock (MC398)-associated MRSA.

Based on a novel algorithm that integrates time, place, and genetic distance, 1724 isolates (27%) formed 155 significant clusters that were unlikely to have occurred by chance (Supplementary Table II; MC8 shown as example in Figure 2). Sixty-eight clusters (44%) included isolates that originated from more than one laboratory, and consequently also from more than one healthcare institution; 76% (1315/1724) of all clustered isolates were part of multi-institutional clusters. The largest cluster included 167 MC8 isolates from 21 different laboratories over 31 months. The proportion of isolates that formed clusters differed considerably between different MLVA clonal complexes. Only 4.4% (52/1195) of the MC398 isolates were part of a cluster in contrast to 47% (285/611) in MC45 (Figure 3). The dominant community- and animal-associated MLVA complexes (i.e. MC8 and MC398) showed lower proportions of clustered isolates than MLVA complexes typically associated with HA-MRSA. The jack-knife analysis did not result in large differences in proportions of clustered isolates for the dominant MLVA clonal complexes (Figure 3). The algorithm thus seems to be robust against missing data, which might arise in the surveillance system or through exclusion in our analysis. Furthermore, the proportion of clustered isolates did not substantially differ when analysed for single years (Supplementary Figure 1), although the confidence bounds increased due to the smaller sample size per MLVA complex/time-frame. The proportion of clustered isolates for MC398 was lower than for any of the other major MLVA complexes.

### Isolates lacking risk factors

Of the 6295 isolates, 2966 (47%) lack obvious risk factors based on the epidemiological data. These consisted of 1405 isolates marked as unexpected findings, from patients without known risk factors, and 1561 clinical isolates submitted directly from laboratories unaccompanied by epidemiological information collected by infection control nurses. Of all these isolates, 579 (19.5%) were part of a cluster, and the proportion of clustered isolates did not differ between the unexpected findings (276/1405, 19.6%) and isolates without epidemiological information (303/1561, 19.4%). We could therefore not establish any epidemiological association for the remaining 2387 isolates lacking obvious risk factors; these thus represent true MUOs and made up 38% of all submitted isolates.

### Epidemiological validation

**Figure 4:**
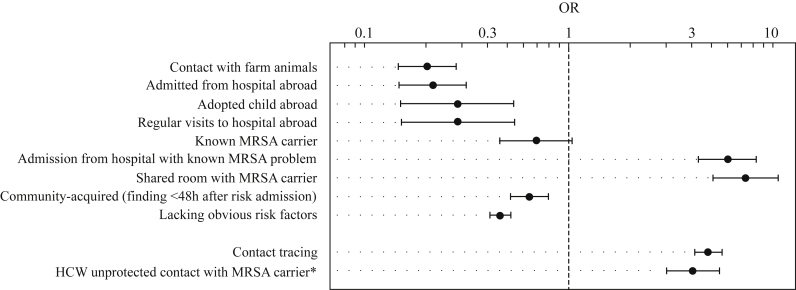
The odds of belonging to a cluster of isolates differ considerably between epidemiological risk categories. The results of the clustering algorithm overlap with expectations based on risk group. The isolates lacking obvious risk factors more often fall outside the clusters, whereas isolates obtained as part of contact tracing during outbreaks were more often found within clusters. This is a healthcare worker (HCW)-specific risk group. OR, odds ratio; MRSA, meticillin-resistant *Staphylococcus aureus*.

For 1757 (28%) isolates, epidemiological metadata including the patient's WIP risk categories were known. The most frequent risk category was ‘contact with farm animals’, which coincided with the isolation of an MC398 strain. Among the other MCs the most frequent risk categories were ‘admission from a foreign hospital’ and ‘admitted to hospital with known MRSA problem’ (Supplementary Table II). The results from the cluster analysis were largely supported by this additional information. Isolates from patients who were admitted after hospitalization abroad were preferentially found outside clusters [odds ratio (OR): 0.22; 95% confidence interval: 0.15–0.31] (Figure 4), with only 8% (31/389) clustered, whereas isolates from patients who shared a room with known MRSA carriers were mainly found within clusters (7.35; 5.09–10.6). Moreover, most isolates (59%, 491/836) identified as part of contact screening were part of a cluster (Supplementary Table II).

**Figure 5:**
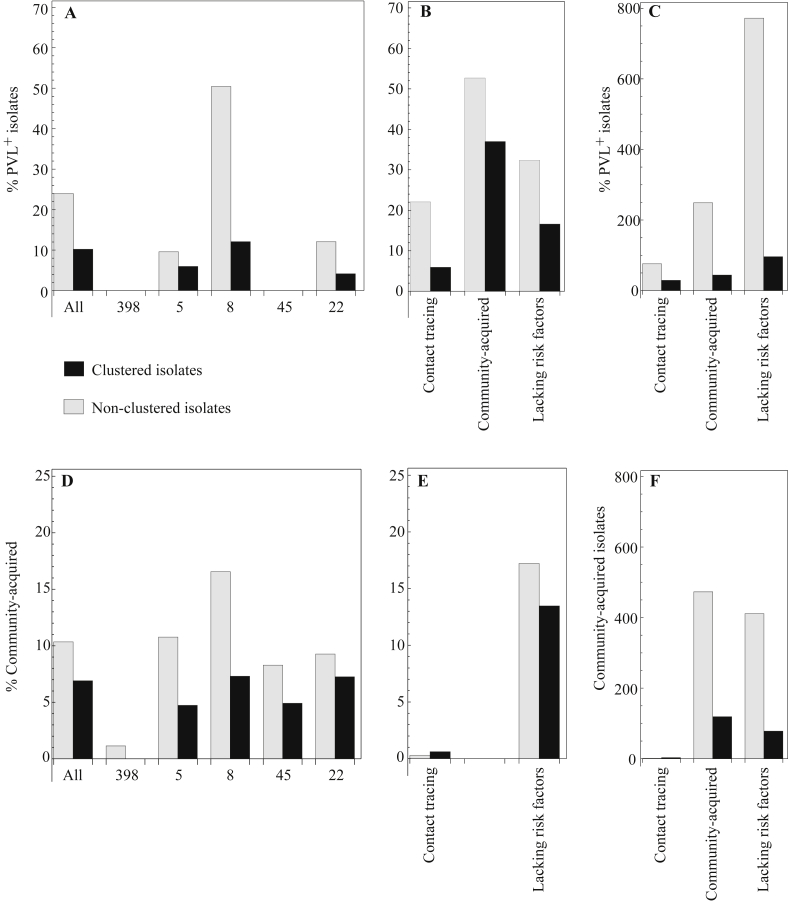
The proportion of isolates positive for Panton–Valentine Leukocidin (PVL) and the proportion of community-acquired (CA) isolates are higher among cases outside the clusters. Black bars show clustered isolates; grey bars show non-clustered isolates. (A) The proportion of PVL-positive cases for the largest MLVA (multi-locus variable number of tandem repeat analysis) clonal complexes (MCs), and (B) for isolates obtained as part of contact tracing during outbreaks, those isolated within 48 h after admission (CA), and without apparent risk factors. (C) In absolute number of isolates, most PVL-positive isolates are found among isolates without apparent risk factors outside clusters. (D) The proportion of CA meticillin-resistant *Staphylococcus aureus* (MRSA) for the largest MCs, and (E) for isolates obtained as part of contact tracing during outbreaks and isolates without apparent risk factors. (F) In absolute number of isolates, most CA-MRSA isolates are found outside clusters.

Isolates assigned as community-acquired MRSA, found within 48 h after admission, were mostly without obvious risk factors (83%, 489/592), and a small proportion of these CA-MRSA isolates clustered (20%, 119/592). Isolates containing the PVL gene, widely associated with community acquisition and mainly representing findings without apparent risk factors in our sample (68%, 868/1271), only clustered occasionally (Figure 5).^9^, ^10^, ^11^ MC8 harboured the largest proportion (41%, 515/1271) of the PVL-positive isolates. Among clustered MC8 isolates, only 12% (43/356) were PVL positive versus 50% (472/936) of those that did not cluster (*P* < 0.0001).

### Healthcare network position

When using the position of the healthcare institutions within the national patient referral network instead of postal code of patients' residence, we identified 1420 isolates (23%) (Supplementary Table III) forming 142 clusters, whereby 47 clusters were multi-institutional. Clusters in this analysis largely overlapped with that using patients' postal code, i.e. 1120 isolates clustered in both analyses, and the proportions of clustered isolates per MLVA clonal complex show a similar pattern in both analyses (Supplementary Figure 2). Of the 2966 isolates without apparent risk factors, 467 (16%) were identified as part of a cluster using this analysis.

## Discussion

Stringent screening policies are implemented in The Netherlands for patients who belong to risk categories. These risk categories identify many MRSA carriers on admission to Dutch hospitals, preventing further nosocomial spread and limiting the number of hospital-acquired MRSA cases. However, a considerable number of MRSA isolates submitted to the RIVM are isolated from patients who do not belong to any of the risk categories, marked as unexpected findings, or were submitted to the reference laboratory without accompanying epidemiological information. Some of these isolates are the result of unobserved transmission whereas others were introduced from hitherto undefined reservoirs. Isolates of the last group may be regarded as MUOs.^4^

Using an integrated clustering algorithm, we mapped the distribution of MLVA types over time and space across the entire Dutch healthcare system, and thus determined the size and clonal composition of clusters of related isolates.^6^ Although the algorithm cannot distinguish between transmission chains inside and outside the hospital, it is far more likely to detect hospital transmission, because isolates were collected in healthcare institutions. Potential transmission chains in the community will inevitably be diluted due to the lower sampling density. This allowed us to infer the proportion of isolates belonging to any specific lineage that is typically associated with intra- and inter-hospital transmission, and to determine the number and genetic diversity of MUOs as an estimate of the frequency of primary introductions and the size of extramural reservoirs.

The algorithm assigned 579 of the 2966 (19.5%) unexpected isolates and isolates without epidemiological information to clusters, indicating a close epidemiological association, probably attributable to transmission. This finding suggests that local hospital epidemiologists are unable to identify all nosocomial transmission events, especially if transmission chains extend over different healthcare institutions. The remainder of these isolates, which represented almost 40% of all included submissions, showed no epidemiological associations and represented true MUOs. Their exact reservoir remains elusive, although the high proportion of PVL-positive isolates among the MUOs in MLVA clonal complex 8 (MC8) suggests a community origin.^9^, ^10^, ^11^ Intriguingly, more of the isolates were assigned to clusters when patients' residential postal codes were used as a proxy for location rather than the position within the national referral network of the patient's healthcare institution. This may be an indication of community transmission and suggests that MUOs recovered from admitted patients indeed represent a sample of MRSA co-circulating among non-hospitalized individuals in Dutch communities.

The shortcomings in the epidemiological data reflect how conventional epidemiological methods rely heavily on data collected on-site by infection control staff. Although the data collection for a single case may appear rather manageable, the cumulative effort for all cases becomes daunting due to the current constraints on human personnel and economic resources in healthcare institutions. This reduces the sensitivity of local investigational epidemiology because cases will be missed and direct links disappear. The autonomous nature of the clustering algorithm is therefore one of its key advantages. The algorithm only requires the most basic information ‒ time, place, and genomic profile ‒ and may suggest epidemiological associations in an automatic manner.

We tested the results for epidemiological consistency using the available metadata for a subset of isolates and found a high correlation between the WIP risk groups and the degree of clustering as suggested by the algorithm. Isolates that were generally considered to be introduced by patients after a period of hospitalization abroad were unlikely to form clusters (OR: 0.22), whereas isolates obtained as part of contact tracing during outbreaks were more often found within clusters (OR: 4.38). However, the sensitivity of clustering algorithms always depends on the sampling density. If fewer isolates are collected, it will become harder for the algorithm to distinguish outbreaks from the independent introductions, because fewer isolates from the outbreak will be available. This would mainly apply to short transmission chains; larger events will still be detected, because the chance of finding two related cases increases with the number of cases in the chain.^7^ It is therefore possible that some of the isolates assigned as independent introductions were still part of small, but largely unobserved, transmissions which have been missed.

We used an algorithm designed to find clusters of infectious disease transmission, as it focuses on chains of cases rather than cases that cluster around a common point (Kulldorff SaTScan™ 2005) in time, space, or genetics.^6^ Because it uses an ordinal distance for each variable, counting the number of isolates between two isolates in question, it does not need to make assumptions on how distances in different variables need to be combined. The algorithm thus tries to find clusters of cases without prior knowledge of the structure of the data. Consequently, some of the links within a cluster may be weak if two of the three epidemiological parameters (location, time, and genetic distance) suggest a close association. For instance, a cluster may include isolates based on spatial and temporal proximity that for evolutionary reasons would be deemed less likely. This is possible in larger clusters, because the algorithm will then allow for weaker links to be included in the cluster, resulting in a slight overestimation of the cluster size or joining of multiple ‘true’ clusters. Due to these potential shortcomings in specificity we expect that the 38% MRSA of unknown origin among all incident MRSA cases in Dutch healthcare institutions between 2008 and 2011 is a conservative estimate.

Using a cluster algorithm to identify clusters of related isolates delivers novel insights in the dynamics of MRSA at national level that can aid the design of novel intervention strategies and guidelines. First, many of the clusters connect multiple healthcare institutions, which would have remained undetected if the isolates were typed only locally and isolates were not made available to reference laboratories. These multi-institutional clusters are likely caused by referred MRSA-positive patients between hospitals.^7^, ^12^, ^13^ It shows how the occurrence of nosocomial pathogens in hospitals should not be viewed as events in single, independent units. Any intervention strategy or surveillance system adds value if it is organized at regional or national level.

Second, the proportion of clustered isolates reflects the propensity of a clone to spread through the hospital population, as nosocomial transmission will result in clustered isolates. The isolates belonging to MC398, representing ST398, show very few clusters. These livestock-associated MRSA isolates are considered to spread easily within and between animal herds, but apparently they only spread through the human population on a very limited scale.^14^, ^15^, ^16^ Most of the MC398 isolates would therefore be the result of independent introductions presumably from the animal reservoir. This same pattern is seen in MRSA strains associated with community acquisition. Novel intervention strategies will require rapid identification at clonal level to take the differential transmissibility into account and inform infection control measures on the basis of epidemic potential of individual clonal lineages of MRSA.

The results also hold implications for the current guidelines in The Netherlands. The search-and-destroy policy is able to prevent a significant part of the MRSA transmission in Dutch hospitals by identifying patients at risk. Thus, the policy contributes to the low MRSA prevalence in the Dutch hospitals, and it should therefore be maintained. However, this will become an increasingly difficult task, as we expect the number of MUOs to rise. The suggested community reservoir of these MUOs calls for a more detailed risk analysis to improve the WIP categories and maintain the low MRSA prevalence in Dutch hospitals.

The implications reach further than the borders of The Netherlands, and show that a national perspective is imperative to understand the dispersal of high risk micro-organisms, such as ‒ but not exclusively ‒ MRSA. Information on the epidemiological and genetic background of cases needs to be collected in central databases, because the dispersal of these clones takes place beyond single healthcare institutions. Making individual hospitals responsible for the reporting of outbreaks will not yield the information needed to correctly inform control policy.

## Conflict of interest statement

None declared.

## Funding sources

This research was funded by the National Institute for Public Health and the Environment (RIVM) strategic research budget (SOR Grant S210026) and the UK Clinical Research Collaboration Translational Infection Research Initiative supported by the Medical Research Council (Grant G1000803). The funding sources had no role in the design of the study or the decision to submit it for publication.
