# Supplementary material for: Monitoring the spread of meticillin-resistant Staphylococcus aureus in The Netherlands from a reference laboratory perspective
Source: J Hosp Infect. 2016 Aug;93(4):366–74. doi: 10.1016/j.jhin.2016.02.022 (PMC4964845; doi:10.1016/j.jhin.2016.02.022)
Supplement: Supplementary file 1 [file mmc1.pdf]

Caring for the bigger picture: Monitoring the national spread of MRSA from a reference laboratory perspective.
-
Supplementary information

Tjibbe Donker Thijs Bosch Rolf Ypma Anja Haenen
Marijn van Ballegooijen Max Heck Leo Schouls Jacco Wallinga
Hajo Grundmann

Table S1: The number of isolates, split up by MLVA clonal complex (MC) and epidemiological information, such as the risk groups defined by the Workgroup Infection Prevention (WIP).

	All*	MC398*	MC5	MC8	MC45	MC22	MC30	MC621	MC80	MC1	MC482	MC2	MC88	MC435	MC7	MC2236	MC632
All	6295	1195	1416	1292	611	549	349	211	201	137	90	63	51	46	31	27	26
WIP Introductions	1064	507	158	122	49	86	26	41	16	20	7	12	4	3	9	2	2
WIP Transmission	522	13	207	109	64	69	13	7	1	8	2	20	2	1	2	0	4
Without obvious risk factors	2966	434	659	689	253	238	220	100	135	74	54	10	28	30	15	14	13
Contact Tracing	836	21	198	215	174	81	38	21	18	16	11	18	7	3	3	7	5
Conflicting information	907	220	194	157	71	75	52	42	31	19	16	3	10	9	2	4	2
Contact with Farm Animals	*543*	*497*	*12*	*8*	*7*	*4*	*4*	*4*	*3*	*1*	*2*	*0*	*0*	*0*	*0*	*1*	*0*
Admitted from foreign hospital	*389*	*7*	*109*	*87*	*37*	*80*	*19*	*6*	*13*	*9*	*3*	*12*	*3*	*3*	*0*	*0*	*1*
Adopted child	*112*	*2*	*29*	*25*	*2*	*2*	*2*	*31*	*0*	*9*	*0*	*0*	*1*	*0*	*9*	*0*	*0*
>2 months ago in foreign hospital	*22*	*1*	*11*	*2*	*3*	*0*	*1*	*0*	*0*	*1*	*1*	*0*	*0*	*0*	*0*	*1*	*1*
Foreign dialysis patient	*3*	*0*	*2*	*0*	*0*	*0*	*0*	*0*	*0*	*0*	*1*	*0*	*0*	*0*	*0*	*0*	*0*
Protected contact with MRSA carrier	*21*	*2*	*4*	*6*	*1*	*7*	*0*	*0*	*0*	*0*	*0*	*1*	*0*	*0*	*0*	*0*	*0*
Unprotected contact with MRSA carrier	*194*	*5*	*77*	*34*	*30*	*21*	*10*	*2*	*1*	*2*	*0*	*5*	*2*	*1*	*0*	*0*	*4*
Admitted to hospital with known MRSA problem	*178*	*5*	*81*	*48*	*14*	*18*	*2*	*1*	*0*	*5*	*1*	*3*	*0*	*0*	*0*	*0*	*0*
Shared room with MRSA patient	*152*	*4*	*55*	*27*	*22*	*23*	*1*	*4*	*0*	*1*	*1*	*12*	*0*	*0*	*2*	*0*	*0*
Known MRSA carrier	*149*	*20*	*46*	*29*	*11*	*12*	*13*	*5*	*6*	*2*	*1*	*0*	*1*	*0*	*0*	*2*	*1*
Known HCW MRSA carrier	*5*	*0*	*0*	*2*	*1*	*1*	*0*	*0*	*0*	*0*	*0*	*0*	*0*	*1*	*0*	*0*	*0*

Table S2: The number of isolates assigned to a cluster using the postal code of the patient s residential address as location data, split up by MLVA clonal complex (MC) and epidemiological information, such as the risk groups defined by the Workgroup Infection Prevention (WIP).

	All*	MC398*	MC5	MC8	MC45	MC22	MC30	MC621	MC80	MC1	MC482	MC2	MC88	MC435	MC7	MC2236	MC632
All	1724	52	571	356	285	193	35	49	37	37	36	21	21	13	3	10	5
WIP Introductions	94	33	23	12	8	9	1	3	0	1	1	1	0	0	0	1	1
WIP Transmission	342	1	145	73	37	57	4	7	0	6	1	8	0	1	2	0	0
Without obvious risk factors	579	10	194	109	94	55	9	16	28	21	17	1	10	6	1	7	1
Contact Tracing	491	1	139	122	113	56	12	10	5	5	4	11	7	1	0	2	3
Conflicting information	222	10	70	40	34	16	9	13	4	4	13	0	4	5	0	0	0
Contact with Farm Animals	*46*	*33*	*5*	*3*	*3*	*0*	*0*	*2*	*0*	*0*	*0*	*0*	*0*	*0*	*0*	*0*	*0*
Admitted from foreign hospital	*31*	*0*	*12*	*4*	*4*	*9*	*1*	*0*	*0*	*0*	*0*	*1*	*0*	*0*	*0*	*0*	*0*
Adopted child	*11*	*0*	*4*	*5*	*0*	*0*	*0*	*1*	*0*	*1*	*0*	*0*	*0*	*0*	*0*	*0*	*0*
>2 months ago in foreign hospital	*7*	*0*	*4*	*0*	*1*	*0*	*0*	*0*	*0*	*0*	*0*	*0*	*0*	*0*	*0*	*1*	*1*
Foreign dialysis patient	*2*	*0*	*1*	*0*	*0*	*0*	*0*	*0*	*0*	*0*	*1*	*0*	*0*	*0*	*0*	*0*	*0*
Protected contact with MRSA carrier	*10*	*1*	*1*	*4*	*0*	*4*	*0*	*0*	*0*	*0*	*0*	*0*	*0*	*0*	*0*	*0*	*0*
Unprotected contact with MRSA carrier	*115*	*0*	*49*	*24*	*14*	*18*	*3*	*2*	*0*	*1*	*0*	*3*	*0*	*1*	*0*	*0*	*0*
Admitted to hospital with known MRSA problem	*121*	*0*	*61*	*31*	*10*	*13*	*1*	*1*	*0*	*4*	*0*	*0*	*0*	*0*	*0*	*0*	*0*
Shared room with MRSA patient	*110*	*0*	*42*	*18*	*15*	*22*	*0*	*4*	*0*	*1*	*1*	*5*	*0*	*0*	*2*	*0*	*0*
Known MRSA carrier	*31*	*1*	*15*	*4*	*5*	*2*	*0*	*1*	*1*	*0*	*1*	*0*	*1*	*0*	*0*	*0*	*0*
Known HCW MRSA carrier	*2*	*0*	*0*	*2*	*0*	*0*	*0*	*0*	*0*	*0*	*0*	*0*	*0*	*0*	*0*	*0*	*0*

Table S3: The number of isolates assigned to a cluster using the position of the sending hospital laboratory in the patient referral network as location data, split up by MLVA clonal complex (MC) and epidemiological information, such as the risk groups defined by the Workgroup Infection Prevention (WIP).

	All*	MC398*	MC5	MC8	MC45	MC22	MC30	MC621	MC80	MC1	MC482	MC2	MC88	MC435	MC7	MC2236	MC632
All	1420	4	462	317	265	162	39	24	24	35	28	12	21	18	3	0	6
WIP Introductions	68	1	16	9	12	13	2	2	0	2	2	5	1	2	0	0	1
WIP Transmission	270	0	114	60	23	52	5	6	0	5	1	1	0	1	2	0	0
Without obvious risk factors	467	1	161	92	84	36	12	8	18	19	12	4	9	8	1	0	2
Contact Tracing	462	2	123	129	121	49	10	5	5	3	4	0	7	1	0	0	3
Conflicting information	119	0	40	21	17	7	8	3	1	4	9	0	4	5	0	0	0
Contact with Farm Animals	*15*	*1*	*4*	*2*	*4*	*1*	*1*	*2*	*0*	*0*	*0*	*0*	*0*	*0*	*0*	*0*	*0*
Admitted from foreign hospital	*46*	*0*	*10*	*4*	*8*	*12*	*1*	*0*	*0*	*1*	*2*	*5*	*1*	*2*	*0*	*0*	*0*
Adopted child	*6*	*0*	*3*	*2*	*0*	*0*	*0*	*0*	*0*	*1*	*0*	*0*	*0*	*0*	*0*	*0*	*0*
>2 months ago in foreign hospital	*2*	*0*	*0*	*1*	*0*	*0*	*0*	*0*	*0*	*0*	*0*	*0*	*0*	*0*	*0*	*0*	*1*
Foreign dialysis patient	*1*	*0*	*1*	*0*	*0*	*0*	*0*	*0*	*0*	*0*	*0*	*0*	*0*	*0*	*0*	*0*	*0*
Protected contact with MRSA carrier	*11*	*0*	*1*	*4*	*0*	*6*	*0*	*0*	*0*	*0*	*0*	*0*	*0*	*0*	*0*	*0*	*0*
Unprotected contact with MRSA carrier	*85*	*0*	*37*	*18*	*7*	*16*	*3*	*1*	*0*	*1*	*0*	*1*	*0*	*1*	*0*	*0*	*3*
Admitted to hospital with known MRSA problem	*98*	*1*	*47*	*26*	*8*	*9*	*2*	*1*	*0*	*4*	*0*	*0*	*0*	*0*	*0*	*0*	*0*
Shared room with MRSA patient	*90*	*0*	*36*	*16*	*10*	*21*	*0*	*4*	*0*	*0*	*1*	*0*	*0*	*0*	*2*	*0*	*0*
Known MRSA carrier	*22*	*0*	*12*	*2*	*5*	*2*	*0*	*0*	*0*	*0*	*0*	*0*	*1*	*0*	*0*	*0*	*0*
Known HCW MRSA carrier	*2*	*0*	*0*	*2*	*0*	*0*	*0*	*0*	*0*	*0*	*0*	*0*	*0*	*0*	*0*	*0*	*0*

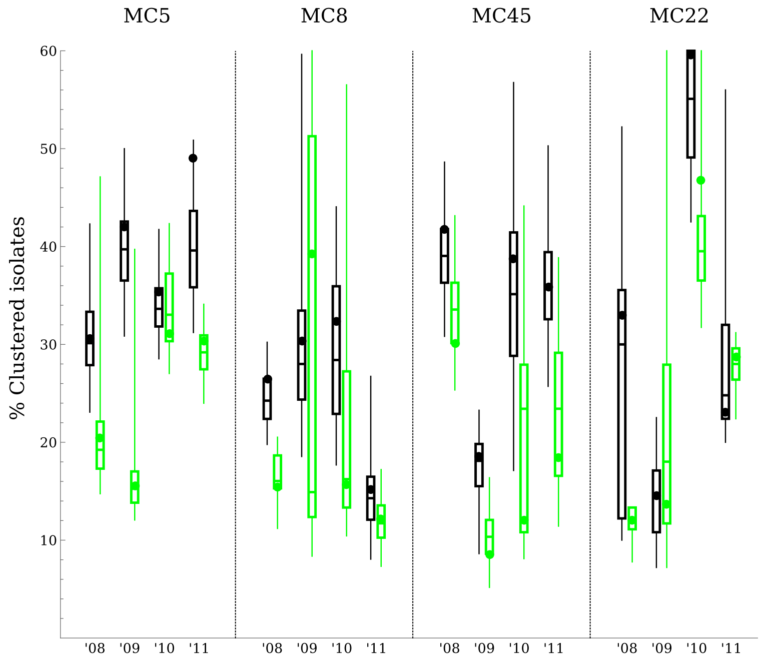

Figure S1: **The proportion of isolates part of a cluster according to the algorithm per year for the major MLVA complexes.** The results of the analysis using the postal code are shown in black, results from the analysis using the patient referral network in green. Using single years of data increases the uncertainty in the estimate, but does not cause a substantial reduction in the proportion clustered isolates.

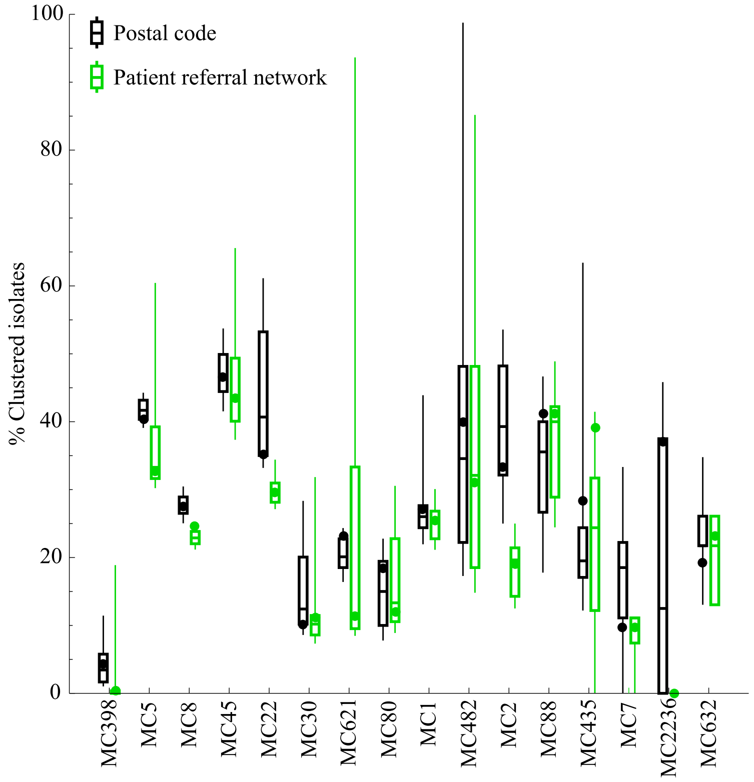

Figure S2: **The proportion of isolates part of a cluster according to the algorithm.** The results of the analysis using the postal code are shown in black, results from the analysis using the patient referral network in green. Using the network as distance measure generally delivers less and smaller clusters.

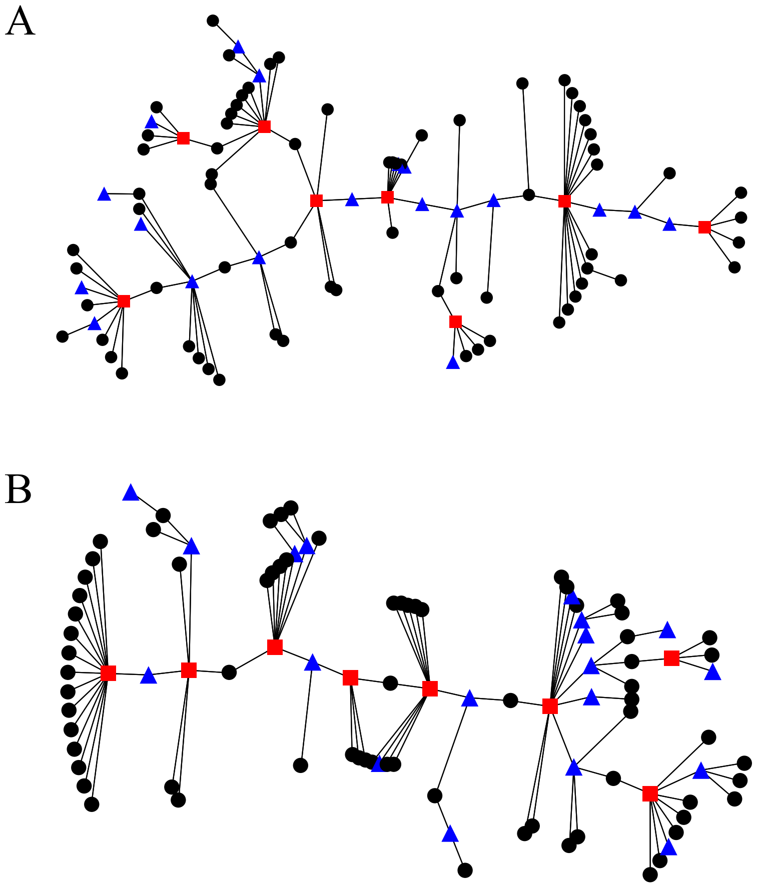

Figure S3: **The structure of the patient referral network shown as a minimum spanning tree.** A) The patient referral network constructed from the Dutch Medical Registry 2004. B) The network reconstructed using geographical distances between hospitals, weighted by a hospital category/specific factor. Red squares denote university hospitals, blue triangles teaching hospitals, and black dots general hospitals. The structure of both networks is more or less the same, with groups of general hospitals around university hospitals, and a back/bone structure consisting of mostly University and teaching hospitals.

Simulating the hospital network

In order to measure the distance between hospitals, we simulated the national patient referral network of the Netherlands. The general structure of this network [1] is known, however, the exact position of each of the hospitals is not known, because they are only known by their semi/anonymous identifier. We therefore calculated the pairwise distance between the coordinates of all hospitals, measured on the Dutch national grid. These distances (D_{ij}) were then used to calculate the link strength between hospitals:

$$L_{ij} = w_{ij}/D_{ij}$$

Where w_{ij} is a weight factor (See table S4) to compensate for the disassortative mixing between hospital categories. The resulting metric is similar to the patient flow between hospitals, a high value indicates hospitals close together, a low value hospitals further apart. It is, however, not a direct reflection of the number of exchanged patients, but the resulting network has a similar structure to the original measured one (Figure S3).

References

[1] Donker T, Wallinga J, & Grundmann H (2010) Patient referral patterns and the spread of hospital-acquired infections through national health care networks. *PLoS computational biology*, **6**(3): e1000715.

Table S4: To simulate the patient referral network, the geographical distance between hospitals was compensated by a weight depending on the types of hospitals, to account for the disassortative mixing between hospitals in the network (i.e. general hospitals tend to refer to teaching or university hospitals, not to other general hospital).

	General	Teaching	University
General hospital	1	3	5
Teaching hospital	3	1	7
University hospital	5	7	1
